# Computational modeling and *in silico *analysis of differential regulation of *myo*-inositol catabolic enzymes in *Cryptococcus neoformans*

**DOI:** 10.1186/1471-2199-9-88

**Published:** 2008-10-14

**Authors:** Emalee A Mackenzie, Lisa S Klig

**Affiliations:** 1 Life Sciences Department, Irvine Valley College, Irvine, CA, USA; 2 Department of Biological Sciences, California State University, Long Beach, CA, USA

## Abstract

**Background:**

Inositol is a key cellular metabolite for many organisms. *Cryptococcus neoformans *is an opportunistic pathogen which primarily infects the central nervous system, a region of high inositol concentration, of immunocompromised individuals. Through the use of *myo*-inositol oxygenase *C. neoformans *can catabolize inositol as a sole carbon source to support growth and viability.

**Results:**

Three *myo*-inositol oxygenase gene sequences were identified in the *C. neoformans *genome. Differential regulation was suggested by computational analyses of the three gene sequences. This included examination of the upstream regulatory regions, identifying ORE/TonE and UAS_INO_ sequences, conserved introns/exons, and in frame termination sequences. Homology modeling of the proteins encoded by these genes revealed key differences in the *myo*-inositol active site.

**Conclusion:**

The results suggest there are two functional copies of the *myo*-inositol oxygenase gene in the *C. neoformans *genome. The functional genes are differentially expressed in response to environmental inositol concentrations. Both the upstream regulatory regions of the genes and the structure of the specific proteins suggest that *MIOX1 *would function when inositol concentrations are low, whereas *MIOX2 *would function when inositol concentrations are high.

## Background

*Myo*-inositol, a key cellular metabolite, is a simple six carbon ring sugar with one hydroxyl group on each carbon. *Myo*-inositol is the precursor for the synthesis of phosphatidylinositol, an essential membrane lipid, an anchor for proteins, and a core component of signal transduction mechanisms [1,2]. Inositol and compounds derived from inositol are among the major nonperturbing intracellular osmolytes which accumulate in response to hypertonic stress of the organism or tissue. Stepwise phosphorylation of inositol yields the *myo*-inositol polyphosphates. Inositol hexakisphosphate, in particular, has been found in soil, bacteria and most animals [2].

*Cryptococcus neoformans *is an opportunistic pathogen primarily infecting individuals with compromised immune systems. *C. neoformans *is found worldwide in soil and pigeon droppings. Under environmentally dry conditions, the quiescent fungal spores in the soil or pigeon guano can become airborne. Once in the air mammals can inhale the dehydrated yeast spores. The immune system of a non-immunocompromised individual typically eliminates *C. neoformans *with out any symptoms of disease. If the host is immunocompromised the pathogen can cause cryptococcosis. *C*. *neoformans *infections often localize to the brain and central nervous system (CNS) [3]. *C*. *neoformans *is unusual among the fungi in that the pathogen can use inositol as a sole carbon source to support growth. Catabolism of *myo*-inositol in *C*. *neoformans *is through the action of *myo*-inositol oxygenase (MIOX), though this has not been confirmed as the only pathway [4]. The inositol concentration in the cerebral spinal fluid (CSF) is high (when compared to plasma levels [5]). *C*. *neoformans *localized in the CNS could utilize *myo*-inositol as a substrate for *myo*-inositol oxygenase (MIOX) in order to generate glucuronic acid for energy production.

The conversion of *myo*-inositol to glucuronic acid by MIOX involves the cleavage of the inositol ring between the 6 C and 1 C, and a four-electron transfer with 1 atom of oxygen incorporated into the glonate, xylulose and xyulose-5-phosphate which can then enter the pentose phosphate pathway resulting in energy production.

This enzyme has been extensively studied in many eukaryotic organisms, recently crystallized, and modeled [6-9]. However, regulation of the MIOX protein in any organism has only recently been examined. Transcription of the *MIOX *gene in humans has been shown to respond to osmotic response element (ORE) binding proteins and/or the tonicity-responsive enhancer (TonE) binding protein through conserved motifs [7,10]. Expression of genes regulated by ORE/TonE binding proteins have been shown to increase when an AP-1 protein binding sequence is located downstream. In conditions of high osmolarity, this AP-1 mediated increase in transcription is inhibited by A-Fos or Tam-67 [11]. The promoter of a renal specific oxidoreductase with increased expression in diabetes mellitus, that has been experimentally determined to respond to inositol in media, also contains the conserved ORE motif GGAAA [6]. An additional transcriptional regulating sequence, known as an inositol upstream activation sequence (UAS_INO_), has the conserved core sequence of CANNTG and has been identified upstream of several genes encoding proteins involved in phospholipid metabolism [1].

In this study, three genes on separate chromosomes, encoding the MIOX protein, were identified in the *C. neoformans *genome. The MIOX promoter region, transcriptional regulatory sequences and *myo*-inositol binding pocket in *C. neoformans *were characterized. Examination of the genes revealed differential regulation. This examination includes identification of upstream regulatory sequences such as ORE, UAS_INO_, and TATA boxes, introns, in-frame termination sequences, expressed sequence tags (ESTs) and CpG islands for each sequence. Molecular modeling of the three protein sequences indicated key differences between the isoforms possibly affecting the ability of two isoforms to bind the *myo*-inositol substrate.

## Results

### MIOX Gene Identification and Characterization

**Figure 1:**
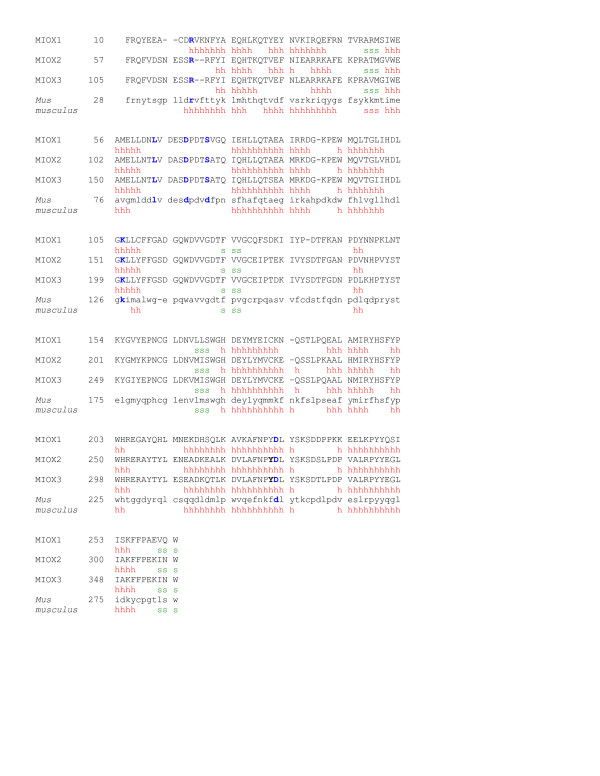
**Optimal alignment of *C. neoformans myo*-inositol oxygenase to the *Mus musculus myo*-inositol oxygenase**. MIOX1, MIOX2 and MIOX3 refers to the *C. neoformans myo*-inositol oxygenase protein isoforms. The predicted consensus secondary structure for each sequence is below the respective amino acid. Helices are represented by h and are in red. Sheets are represented by s and are in green. Each protein is predicted to have 10 helices and four sheets. Conserved amino acids essential to enzyme function are highlighted in blue.

Examination of the STGC *C. neoformans *genomic database using BLAST identified a sequence within chr06.b3501.040616 that is a 100% match to the experimentally determined N-terminal sequence [12] of the *C. neoformans *MIOX protein (Figure 1). The sequence was extracted along with an additional 3000 bases upstream and downstream. A keyword search of the TIGR *C. neoformans *computationally annotated database revealed three possible MIOX genes on separate chromosomes. TIGR sequence 180.m00186 mapped to chromosome 6, 189.m00292 mapped to chromosome 8 and 177.m03138 mapped to chromosome 7. Sequence 180.m00186 contains the experimentally determined N-terminal region and aligns to contig chr06.b3501.040616 (e-value 0.0) in the STGC *C. neoformans *database. Sequence 189.m00292 aligns with contig chr09.b3501.040506 (e-value 2e-08) and sequence 177.m03138 aligns with chr07.b3501.040506 (e-value 2e-07). The sequence on chromosome 6 (180.m00186) is referred to as *MIOX1*, chromosome 8 (189.m00292) is referred to as *MIOX2 *and on chromosome 7 (177.m03138) is referred to as *MIOX3*.

Computational analysis of the promoter region of *MIOX1 *revealed two possible conserved OREs containing the consensus sequence GGAAA [6]. One of these putative ORE sequences, GGGAAAATTGA, is located at -2137 upstream from the transcriptional start site. Another putative ORE, TGGAAAAAAAGA, is located -645 and is followed by an AP-1 binding sequence (TGATTCA) located at -204. One putative *cis*-acting inositol upstream activating sequences (UAS_INO_) CATGTGGAAT was located at -397, and matches the experimentally determined sequence [13](Table 1). *MIOX1 *has one predicted TATA box starting at nucleotide -106. EST b9fo8h9.r1 in the TIGR database aligned with bases -87–229, 280–455 with a 95% identity. EST a7e05cn.r1 aligned with nucleotides 540–764 823–947, and 1007–1058 with 93% identity (Table 2). Thus, the *MIOX1 *gene contains three introns with GT/AG splice sites confirmed by comparison of the genomic sequence to ESTs. Four in-frame termination signals were located at the end of the genomic sequence.

**Table 1:** *C. neoformans *Upstream Regulatory Sequences.

Protein	ORE/TonE Sequence location	ORE/TonE sequence	AP-1 Location	AP-1 sequence	UAS_INO _Location	UAS_INO _Sequence
MIOX3	None			None		None

MIOX2	-2714	AGGAAAGCTG		None		None

	-2636	TGGAAAACTG				

MIOX1	-2137	GGGAAAATTGA	-204	TGATTCA	-397	CATGTGGAATT

	-645	GGAAAAAAAGA				

Within 3000 nucleotides up-stream of the *MIOX1 *translation start two ORE/TonE sequences, one AP-1 sequence and one UASINO were identified. Within 3000 nucleotides up-stream of the translation start for *MIOX2 *two ORE/TonE sequences were identified. No consensus ORE/TonE, AP-1 or UASINO sequences were found within 3000 nucleotide sequences upstream of the transcription start site for *MIOX3*.

**Table 2:** Identification of Expressed Sequence Tags That Align with *C. neoformans MIOX *Gene Sequences.

Protein	Expressed sequence Tags (EST)	Nucleotide Match	% identity
MIOX3	None identified		

MIOX2	d4e08j2.r1	-184–37, 86–264, 322–498	98

	a9e06cn.r1	664–846, 900–1107	96

MIOX1	b9fo8h9.r1	-87–229, 280–455	95

	a7e05cn.r1	540–764, 823–947, 1007–1058	93

Two ESTs were identified in the TIGR database for both MIOX1 and MIOX2 with at least 93% identity. No ESTs were found for MIOX3 with an identity above 65%.

The genomic region (TIGR189.m00292 plus +/- 3000 extracted from chr09.b3501.040506 STGC) upstream of the translational start of MIOX2 contains two conserved ORE sequences AGGAAAGCTG and TGGAAAACTG located at -3714 and -3636. Neither sequence was followed by a conserved AP-1 sequence (Table 1). Two ESTs, from the TIGR database were found to align with the coding region of *MIOX2*. EST d4e08j2.r1 aligns to bases -184–37, 86–264 and 322–498 (98%). EST a9e06cn.r1 aligns with nucleotides 664–846, and 900–1107 (96%) (Table 2). Three introns with GT/AG splice sites were confirmed by comparison of the genomic sequence to ESTs. The AG at the 3'end of the splice site of a fourth computationally predicted intron was confirmed by comparison of the genomic sequence to EST d4e08j2.r1. The GT at the 5' end of this intron has not yet been confirmed by EST data. Four in frame termination signals were identified.

Analysis of the genomic region (TIGR 177.m03138 plus 3000 +/- extracted from STGC chr07.b3501.040506) of *MIOX3 *upstream from the transcriptional start site for the *MIOX3 *protein has several computationally identified possible ORE sequences. However, none of the sequences are strictly identical to the experimentally determined ORE sequences and none are followed by a known AP-1 binding site. The *MIOX3 *sequence was determined to contain only one TATA box starting at nucleotide 27 (Table 1). No corresponding ESTs were identified in the TIGR *C. neoformans *database with an alignment better than 65% (Table 2). Computational analysis, along with manual alignment of the coding DNA from TIGR database to the genomic sequence, predicted four introns in *MIOX3*. *MIOX3 *genomic 3'sequence has four in frame termination signals.

*MIOX1 *and *MIOX2 *have a relatively few CpG islands when compared to *MIOX3*. *MIOX3 *has five CpG islands in the upstream region that are approximately evenly spaced and several islands located internal to the gene. The five upstream CpG islands are located at -(51..327), -(752..1006), -(1318..1661), -(1746..2007) and -(2817..3080). *MIOX2 *has 2 CpG islands located from -(457..684), and -(2555..2754). *MIOX1 *has three CpG islands. One island is close to the transcriptional start located at -(48–373). The other two CpG islands identified in the *MIOX1 *gene are further from the start site located at -(2325..2545) and -(2609..2881).

As RNA stabilization can effect functional expression, the predicted RNAs were examined. Secondary and tertiary analysis of the computationally predicted RNA showed no significant variance between the corresponding structures of the three genes (data not shown).

### MIOX Sequence alignment and Homology Modeling

**Figure 2:**
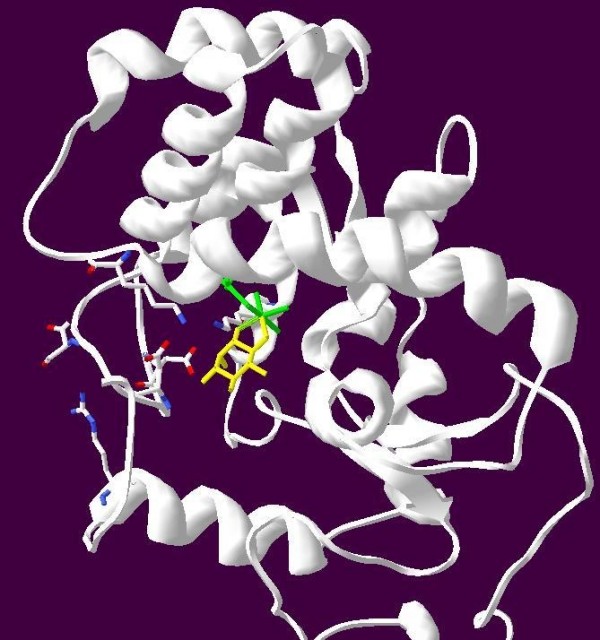
**Computationally Predicted Structure of *C. neoformans *MIOX1**. A. Stereo ribbon diagram, Fe(II) atoms shown in green and inositol in yellow. Key amino acids involved in substrate lid stabilization shown in CPK coloration.

**Figure 3:**
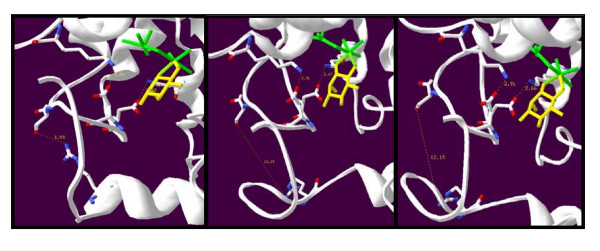
**Computationally Predicted Active Site of the *C. neoformans *MIOX Isoforms**. Bonding distances between the key amino acid side chains involved in the salt bridge stabilization of MI substrate lid. Key amino acids shown in CPK colorization. A: MIOX1 protein. B: MIOX2 protein. C: MIOX3 protein.

In the *Mus musculus *MIOX protein the *myo*-inositol substrate has been determined to be buried in a pocket formed by two short sections of the protein and a hairpin loop [8]. Multiple sequence alignment of the *Mus musculus *MIOX protein sequence and the three MIOX protein sequences from *C. neoformans *reveals 100% identity for the two short sections of the protein (Figure 1). Despite the similarity of the protein sequences and the conservation of several key amino acids, homology modeling of the putative proteins encoded by the three *C. neoformans *genes revealed some significant differences (Figure 2 and 3). The following amino acid numbering is based on the mouse protein [8]. Amino acid Asp-124 was previously identified to be critical for the binding of iron, which is necessary for optimal protein function. This amino acid (Asp-124) is conserved in all three *C. neoformans *MIOX proteins [8]. The hairpin loop consisting of 11 residues (Leu-83, Val-84, Asp-85, Glu-86, Ser87, Asp 88, Pro-89, Asp90, Val-91, Asp-92 Phe-93) in the *Mus musculus *protein has an essential role in binding the *myo*-inositol by forming a lid that closes over the substrate in the active site. This hairpin loop secures the inositol substrate in place (Table 3). The first eight residues in the hairpin loop sequence are strictly conserved in the MIOX1 protein. The next three amino acids of the hairpin loop in the MIOX1 protein (amino acids nine to eleven) differ as follows: Thr for Val-91 replacing a neutral residue with a non-polar, Ser for Asp-92 inserting a neutral residue in place of an acidic residue and Val for Phe-93 both non-polar residues (Table 4, Figure 3). The first three residues (Leu, Val, Asp) and residues five through eight (Ser, Asp, Pro Asp) in the MIOX2 and the MIOX3 predicted hairpin loops align with the experimentally determined hairpin loop identified in the *Mus musculus*. The fourth residue of the hairpin loop has a substitution of Ala for Asp in both the MIOX2 and the MIOX3 proteins, replacing an acidic residue for a smaller non-polar residue. Amino acid residues nine and ten of the hairpin loop are the same as those located in the MIOX1 protein (Thr and Ser). The last residue in the hairpin loop is Phe in the *Mus musculus *MIOX protein, Val in the MIOX1 protein, and Ala in the MIOX2 and MIOX3 proteins.

**Table 3:** Relative Positions of Amino Acids Involved in the Predicted Hairpin Loop Stabilizing the Binding of the *Myo*-Inositol Substrate.

Protein	Amino Acid	Amino Acid	Amino Acid	Amino Acid	Amino Acid	Amino Acid	Amino Acid	Amino Acid	Amino Acid	Amino Acid	Amino Acid
MIOX3	Leu-158	Val-159	Asp-160	Ala-161	Ser-162	Asp-163	Pro-164	Asp-165	Thr-166	Ser-167	Ala-168

MIOX2	Leu-110	Val-111	Asp-112	Ala-113	Ser-114	Asp-115	Pro-116	Asp-117	Thr-118	Ser-119	Ala-120

MIOX1	Leu-115	Val-116	Asp-117	Glu-118	Ser-119	Asp-120	Pro-121	Asp-123	Thr-124	Ser-124	Val-125

*Mus musculus*	Leu-83	Val-84	Asp-85	Glu-86	Ser-87	Asp-88	Pro-89	Asp-90	Val-91	Asp-92	Phe-93

The hairpin loop is strongly conserved between the four proteins with only a few exceptions: a substitution of Ala for Glu-86, and Ala for Phe-93 in MIOX 2 and MIOX3 proteins, a substitution of Ser for Asp 92 in all three MIOX protein sequences and a conservative substitution of Val for Phe-93 in the MIOX1 protein sequence.

**Table 4:** Amino Acids Involved in Salt-Bridges that Stabilize the *myo*-Inositol Substrate Lid.

Protein	Amino Acid	Distance	Amino Acid	Distance	Amino Acid	Distance
MIOX3	Asp-160/Lys-201	2.67 Å	Asp-163/Lys-331	2.91 Å	Ser-167/Arg-116	12.15 Å

MIOX2	Asp-112/Lys-153	2.66 Å	Asp-115/Lys-283	2.91 Å	Ser-119/Arg-68	12.15 Å

MIOX1	Asp-117/Lys-157	2.65 Å	Asp-120/Lys-287	2.91 Å	Ser-124/Arg-71	3.55 Å

musculus	Asp-85/Lys-127	2.31 Å	Asp-88/Lys-257	2.97 Å	Asp-92/Arg-39	2.98 Å

The amino acids involved in the *myo*-inositol substrate lid are conserved among the four proteins with the exception of a substitution of Ser for Asp in all three computationally modeled *C. neoformans *MIOX proteins. Predicted bonding distances indicate the salt bridge formation is conserved for MIOX1. The distance measured between the substituted Ser and Arg in both computationally modeled MIOX2 and MIOX3 proteins (12.15 Å) does not support the formation of a salt bridge at these locations.

Analysis of the homology models of MIOX2 and MIOX3 proteins in *C. neoformans *indicates three key differences. The distance measured between Asp-112/lys-153 (2.66Å) and Asp-115/lys-283 (2.91Å) in MIOX2 and Asp-160/lys- 201 (2.67Å) and Asp-163/lys-331 (2.91Å) in MIOX3 protein support the formation of salt bridges similar to the *Mus musculus *MIOX protein. However, the distances measured between Ser-119/Arg-68 (12.15Å) in MIOX2 protein and Ser-167/Arg-116 (12.15Å) in MIOX3 protein are too great to support the formation of a salt bridges (Figure 3). The homology models generated for MIOX2 protein and MIOX3 protein support main chain hydrogen bonds with Arg-68 and Gln-163 similar to the *Mus musculus *MIOX. Optimal alignment of the three MIOX proteins with *Mus musculus *indicates a substitution of Val-61 for Thr-32 in the MIOX2 and Val-109 for Thr-32 in the MIOX3 protein. The substitution does not support the formation of a main-chain hydrogen bond at this location for either the MIOX2 or the MIOX3 protein (Table 5).

**Table 5:** Stabilizing Amino Acids Involved in Main-Chain Hydrogen Bonds.

Protein	Amino Acid	Amino Acid	Amino Acid
MIOX3	Val 109	Arg-116	Gln-211

MIOX2	Val-61	Arg-68	Gln-163

MIOX1	Glu-66	Arg-71	Gln-168

musculus	Thr-32	Arg-39	Gln-136

The main chain hydrogen bonds involving Arg and Gln are predicted to be conserved in all four proteins. MIOX2 and MIOX3 protein share a substitution of Val for Thr disrupting the main chain hydrogen bond found at that location in the *Mus musculus *protein. MIOX1 protein has a conservative substitution of Glu for Thr suggesting the preservation of that main chain hydrogen bond.

## Discussion

*C. neoformans *appears to be the only organism in the animal and fungal kingdoms with multiple MIOX genes. Examination of over 60 completed eukaryotic genomes from the animal and fungal kingdoms revealed that if the MIOX gene is present, there is only one highly conserved copy (data not shown). Perhaps the three copies of the *MIOX *gene in the *C. neoformans *genome represents a physiological mechanism for survival in various environmental inositol concentrations.

This computational study suggests there are at least two sequences regulating transcription of the *C. neoformans MIOX *genes, one involves ORE sequences, the other involves UAS_INO _sequences. ORE sequences were originally identified in vertebrates and UAS_INO _sequences were demonstrated in the yeast *Saccharomyces cerevisiae*, but these sequences are present in the *C. neoformans *genome. As a basidiomycete the *C. neoformans *genome has been shown to contain features similar to other yeasts yet its gene organization is more complex resembling higher eukaryotes [14]. Both *MIOX1 *and *MIOX2 *have two ORE/TonE sequences upstream of the genes. MIOX protein expression in humans has been demonstrated to be regulated by ORE/TonE binding proteins [7,10]. In vertebrates, the experimentally demonstrated binding sites for each transcription factor are slightly different. The core sequence TGGAAA is recognized by ORE binding protein, whereas the core sequence GGAAAA is recognized by TonE binding protein (also known as NFAT5[11]). Interestingly, comparison of the *C. neoformans *promoter regions of *MIOX1 *and *MIOX2 *reveals nucleotide differences in the identified ORE/TonE sequences suggesting differential regulation of the two genes. *MIOX1 *has two ORE like sequences with the conserved TGGAAA sequence coupled with an AP-1 binding sequence. This would render MIOX1 subject to A-Fos and Tam-67 inhibition. Two TonE like sequences are located in *MIOX2 *however, no AP-1 sequence was identified. The presence of the TonE and the absence of the AP-1 binding sequences suggests *MIOX2 *is up-regulated in the presence of inositol but is not subject to inhibition by A-Fos or Tam-67 [11]. Unlike MIOX1 and MIOX2, the *MIOX3 *gene lacked a conserved ORE/TonE sequence within 3000 base pairs upstream of the transcriptional start site suggesting *MIOX3 *is not regulated by the ORE or TonE transcription factors.

In addition to the ORE and AP-1 sequences, *MIOX1 *also contains a UAS_INO _sequence. Several *C. neoformans *genes involved in phospholipid and phospholipid precursor biosynthesis (including *MIOX*) have been experimentally found to be regulated by the availability of inositol and choline in the medium [4,12] Studies of the transcriptional control coordinating expression of these genes in *S. cerevisiae *led to the identification of an upstream activating sequence (UAS) with a core conserved sequence of CANNTG [1,15]. The UAS_INO_ has been found to specifically regulate genes via the INO2p/INO4p bHLH transcription factor complex in response to inositol and choline [1]. The UAS_INO_ sequence experimentally demonstrated to serve for transcription activation is CAT(G/A)TGAA(G/A/T)(T/A) [13]. The presence of a UAS_INO _in the upstream region of the *C. neoformans MIOX1 *but not *MIOX2 *or *MIOX3 *genes further suggests that the genes are differentially regulated. Moreover, *MIOX1 *may be active when inositol concentration levels are low.

Differential regulation is further supported by the identification of expressed sequence tags (ESTs) for MIOX1 and MIOX2 but not MIOX3. ESTs identified for both MIOX1 and MIOX2 in this study confirmed the computationally predicted start sites. Furthermore the splice site junctions for MIOX1 and MIOX2 are supported by alignment of ESTs to genomic DNA. ESTs for MIOX3 were not found however, the splice sites computationally predicted for MIOX3 are identical to the splice sites predicted by ESTs alignment for MIOX2. Duplicated nonfunctional genes are less likely to have ESTs [16]. Unexpressed genes, genes expressed at very low levels or only under specific conditions are also less likely to have associated ESTs/cDNAs. Therefore the absence of ESTs for *MIOX3 *suggests either the gene is only expressed under specific conditions or is not expressed at all.

The identity of the *MIOX2 *and *MIOX3 *coding sequences (80%) is significantly less than that of their amino acids (91%). This can be attributed to changes in the wobble position. Manual analysis of the aligned sequences revealed the majority (78%) of the base changes to be in the wobble position. The synonymous changes allowed the identity of the DNA sequences to decrease but did not change most of the amino acids encoded, thereby retaining the protein sequence identity. The conservation of the amino acid sequence despite changes in the DNA suggests that the function of the protein is conserved through selective pressure.

The MIOX substrate, *myo*-inositol, is held in the active site by several key interactions. Three salt bridges created between Asp-85/Lys127, Asp-88/Lys257 and Asp92/Arg-39 form a lid that holds the substrate in the active site, along with main-chain H-bonds with Thr-32, Arg-39 and Gln-136 that stabilize the active site. All residues, essential in *myo*-inositol binding were found to be conserved in *C. neoformans *MIOX1 except Thr-32 and Asp-92. Optimal alignment of the *Mus musculus *and *C. neoformans *MIOX1 protein sequences indicates a conservative substitution of Thr-32 to Glu and Asp-92 to Ser. Thr and Glu are both neutral residues and capable of participating in hydrogen bonds, indicating that the main chain hydrogen bonds found in *Mus musculus *are all conserved in MIOX1. Asp-92 interacts with Arg-39 to form a salt bridge (dist 2.98Å) in the *Mus musculus *MIOX protein. Substitution of Ser for Asp-92 in *C. neoformans *MIOX1 potentially disrupts the salt-bridge formation however; the homology model generated in this study of MIOX1 indicates the distance between the amino acids (3.44Å) supports the formation of a salt bridge at that location despite the substitution.

Protein sequence alignment and the homology models generated for the MIOX2 and MIOX3 proteins of *C. neoformans *also indicate three key differences from the mouse MIOX protein. Unlike the changes in the MIOX1 protein where the active site is preserved, mutations in the MIOX2 and MIOX3 residues suggest disruption of the active site for *myo*-inositol possibly leading to decreased protein activity. Optimal alignment of the mouse MIOX and *C. neoformans *MIOX2 and MIOX3 protein sequences indicates substitutions of Thr-32 to Val and Asp-92 to Ser. The change from Thr to Val disrupts one of the three main-chain hydrogen bonds that helps stabilize the *myo*-inositol lid. The loss of the H-bond between Val and the main chain could be the cause of the increased distance between the Ser and Arg salt bridge (12.15Å) making the formation of a salt bridge at that location beyond the acceptable range. The destabilization of the lid may not allow MIOX2 and MIOX3 proteins to function efficiently.

## Conclusion

Multiple copies of the *MIOX *gene is unique to *C. neoformans *among the animal and fungal kingdoms. This study suggests that the *C. neoformans *genome has multiple copies of the MIOX gene which appear to be differentially expressed under various physiological inositol conditions. MIOX1 protein is predicted to be efficient in binding the *myo*-inositol substrate [12]. *MIOX1 *gene expression is probably active in conditions of low inositol via a UAS_INO_, up-regulated in the presence of inositol, yet possible inhibited by high levels of inositol by A-Fos or Tam-67 at the AP-1 site. This inhibition may be compensated for by the up-regulation of MIOX2. Although the MIOX2 enzyme is not predicted to be as efficient at holding the *myo*-inositol substrate in the active site, due to loss of disulfide bridges between the lid and main-chain and loss of main-chain hydrogen bonds, expression of MIOX2 should not be inhibited in elevated inositol levels due to the absence of an AP-1 binding sequence. This differentiated regulation of inositol catabolism could facilitate the growth and viability of *C. neoformans *in various environments.

## Methods

### MIOX Gene Identification and Characterization

To locate the *MIOX *gene within the *Cryptococcus neoformans *genome the N-terminus region of MIOX1 protein (previously isolated in this laboratory) was submitted as a query search to the TBLASTN program via BLAST at the Stanford Genome Technology Center (STGC). A sequence with 100% identity to the MIOX protein N-terminus region plus 3000 bases +/- was then used to search for cDNA's and ESTs in the TIGR *C. neoformans *gene indices database using BLAST (blastn), and the were aligned with GAP from the Wisconsin sequencing package on the W-H2 server (GCG)[16]. Possible open reading frames (ORF) were located using Map (GCG) Translate (GCG), GENSCAN [17], and ORF Finder [18]. Putative promoter regions were delineated by Promoter Prediction [19], WWW Promoter Scan [20] HCtata [21] Genie [22] and NetStart 1.0 [23] and manual examination. Exon boundaries were predicted using GAP (GCG), NetGene2 [24], Splice Site Prediction [25], Genie [26] and GENSCAN. ORE sequences and UASino sequences were determined by manual analysis of the upstream regions of each MIOX gene sequence. CpG islands were identified using the Vista Genome [27] and European Molecular Biology Laboratory servers [28].

### MIOX Secondary Structure Prediction

Predictions of protein secondary structures for MIOX1, MIOX2, MIOX3 were computed using J-pred [29], PredictProtein [30], PSIPRED [31], Discrimination of protein Secondary structure Class (DSC) [32], Hydrophobic cluster analysis (HCA) [33], PSSFinder [34], and SAM_T02 [35] methods. The results from each method were compared and a consensus structure for each MIOX protein was generated based on regions of similarity. Consensus secondary structure sequences for each MIOX protein were generated by comparing results obtained by all servers, and taking into account only the sequence regions that were predicted at least 50% reliability or higher by at least five servers. All alignments were generated with CLUSTALW [36]or GAP then edited manually using Bioedit [37].

### Template Identification and Protein Modeling

The SWISS-MODEL Comparative Protein Modeling Server [38], along with Modeler within the Accelerys Insight II program suite, were utilized to generate 3D-models of the putative MIOX proteins. The MIOX protein from *Mus musculus *(PDB code: 2HUO) was used as a template. Each *C. neoformans *MIOX protein was manually aligned to the template then submitted to the server. The N-terminal regions of the proteins have a high degree of variation therefore the N-terminal region for each protein was not modeled due to lack of a template. One model was generated in a fully automated way for each *MIOX *sequence based on user defined alignment then verified using PROCHECK [39].

## Authors' contributions

Both EAM, LSK, contributed equally to the research and writing of the manuscript. Both authors read and approved the final manuscript.
